# The Newborn Hearing Screening Programme in Germany

**DOI:** 10.3390/ijns4030029

**Published:** 2018-09-16

**Authors:** Peter Matulat, Ross Parfitt

**Affiliations:** Clinic for Phoniatrics and Paediatric Audiology, University Hospital Muenster, 48149 Münster, Germany

**Keywords:** newborn hearing screening, screening, tracking, follow-up, Germany

## Abstract

This article presents an overview of legal, methodological, organisational, financial, structural and technical aspects of the initial audiological measurement of newborns (screening), follow-up (diagnosis) and tracking the results (tracking) within the German newborn hearing screening programme.

## 1. Introduction

Universal Newborn Hearing Screening (NHS), as defined in the Children’s Directive [1], has been part of the national programme for the screening of infants in Germany since 2009. The objective of the NHS is to enable the diagnosis of hearing impairment at a younger age and thus allow any necessary treatment and rehabilitative care to occur earlier. Early diagnosis poses new challenges, not only for the medical groups involved but also for those involved in the early support of the children concerned.

The aim of this article is to present some legal, methodological, organizational, financial, structural and technical aspects of audiological measurement in newborns (screening), tracking and diagnosis in Germany. The information provided here is based on the standards and protocols specified in the Children’s Directive [1] and the consideration of structures that have been developed before and after the introduction of the NHS in Germany. The screening protocol itself is presented in Section 3. The tracking of findings in need of further monitoring by hearing screening centres (tracking) and specialist medical follow-up (confirmation diagnostics) are presented in separate sections.

## 2. Prevalence

There were 792,131 live births in Germany in 2016 [2]. The final report on the evaluation of the NHS, carried out on behalf of the German Joint Federal Committee [3], estimated a prevalence rate of 1.3 per thousand newborns having permanent bilateral hearing disorders at birth (connatal) (with a hearing loss greater than or equal to 25 dB Hearing Level (HL) and a duration of more than three months). This threshold of 25 dB HL differs from the threshold of 35 dB HL used within screening itself. The 35 dB HL screening threshold reflects the sensitivity threshold of some of the screening equipment and the threshold of 25 dB HL corresponds to the threshold for the specialist medical diagnosis of a hearing disorder. If unilateral hearing disorders are also included, the calculated prevalence rate for congenital hearing disorders in four German federal states in 2009 (after the implementation of universal newborn hearing screening) lay between 1.9 and 2.1 per thousand newborns [4]. We might expect to find one to three children with hearing impairment per thousand livebirths and thus 792 to 2376 people affected in Germany per year, depending on the timepoint of measurement and case definition (i.e., the definition of hearing impairment used to identify cases).

## 3. Newborn Hearing Screening

In a narrow sense, newborn hearing screening is typically understood to consist of using a screening test within the first few days of a newborn’s life. This is a test which attempts to detect responses of the auditory system to auditory stimuli using objective methods with a previously-defined statistical probability. It is usually carried out at the maternity/birth centre.

### 3.1. Programme Objectives and Responsibilities

The Children’s Directive [1] defines the NHS’s primary goals as follows:

- the detection of hearing disorders (separately for each ear) (defined as having hearing thresholds of at least 35 dB HL)
- detection within the first three months of life
- the initiation of therapy before the end of the sixth month of life

This definition is in broad agreement with international guidelines [5]. If a child is born in hospital, the responsibility for carrying out screening lies with the head of the maternity ward. If the birth takes place outside hospital, it is the responsibility of the midwife or doctor accompanying the birth to arrange for hearing screening. Where this does not occur, the family’s paediatrician is obliged to carry out or arrange screening, by the third paediatric examination (U3), which takes place no later than the fifth week of life.

### 3.2. Approved Measurement Techniques

Two automated methods for testing hearing in newborn hearing screening are permitted in Germany: automated auditory brainstem response audiometry (AABR) and automated measurement of transient-evoked otoacoustic emissions (ATEOAE) [1,6].

With ATEOAE the acoustic response emitted by the outer hair cells in the inner ear in response to broadband auditory stimuli are measured by a microphone located in the auditory canal. AABR uses various auditory stimuli to induce electrophysiological activity in the auditory nerve and the brain stem and is detected using electrodes placed on the scalp.

Both test methods are non-invasive and are harmless to the child and his/her hearing. They can be performed on a sleeping or very quiet child. The duration of the measurements themselves, not including preparation time, are on average significantly less than one minute. AABR measurement takes a little longer on average with almost all screening devices, due to the somewhat more complex preparation (mainly attaching the electrodes) and is also slightly more expensive than ATEOAE because of greater consumption of materials (skin abrasive, electrode gel and, if necessary, adhesive scalp electrodes).

Both procedures have a sensitivity of almost 100% (sensitivity is the percentage of newborns with a hearing disorder for whom the hearing disorder is indeed detected). AABR is regarded as the gold standard for the NHS since it has a higher specificity (the probability that newborns with no hearing disorder are indeed recognised as such) than ATEOAE and can also detect retrocochlear causes of hearing impairment (i.e., lesions occurring further up the auditory pathway than the inner ear).

When used for screening, both test techniques are designed to determine whether the probability of an auditory response to a 35 dB HL stimulus being present exceeds a pre-specified statistical likelihood. If successful, a “pass” result is recorded. If proof of an auditory response is not obtained, it is mandatory to undertake a further test using AABR at the same hospital prior to discharge. A lack of auditory response on this further test requires a full diagnostic audiological investigation (“follow-up”) carried out at a specialist paediatric audiology clinic.

A screening result requiring such specialist follow-up in the absence of other risk-factors does not necessarily suggest a high probability of a hearing disorder being present. In practice, a greater number of children fail both of the tests that constitute the NHS than are eventually diagnosed with a hearing loss at the follow-up stage.

### 3.3. Screening Test Procedure

Newborn hearing screening should be performed during the first three days of life and each ear must be tested separately. This prescribed time frame refers to healthy babies born full term. In addition to the two-stage screening test using ATEOAE at first and AABR second, which is common in some countries, the German guidelines [1] also allow the use of an AABR test on two occasions as the NHS. Children at risk of hearing loss [1,7] should always be examined using AABR. Premature babies must be tested no later than the expected date of birth and babies who are ill or have multiple handicaps must be tested by the end of their third month of life. If the initial screen is not successful, it should be repeated or rescreened using AABR, if possible before the mother and child are discharged from hospital. If no auditory response is obtained on both tests, a specialist medical examination with full clinical auditory brainstem response audiometry (ABR) is mandatory. Parents/carers must be informed about this requirement.

Parents/carers should be informed about the medical background of the newborn hearing screening programme and the screening test procedure, and have the right to refuse to have their child screened. The fact that they have been informed of this and the result of the screen should be entered in the child’s personal health record booklet alongside other documented early detection examinations [8].

Measurement can be difficult or even impossible for various reasons, including the presence of vernix caseosa or amniotic fluid in the auditory canal, which can obstruct the conduction of sound to the inner ear. Screening on the second or third day of life gives this fluid chance to clear and is therefore considered an appropriate time frame.

Independent of the evaluation of the measurement quality of the methods used, these legal requirements make it necessary for hospitals to have suitable screening devices (see the overview in Section 3.6. Screening Devices) that can either perform both ATEOAE and AABR measurements or at least perform AABR.

Hearing screening is mainly carried out by nurses or midwives at the maternity/birth centre (see Section 3.8. regarding the responsibilities for hearing screening outside of maternity clinics). Testing may be performed by just one person or many, depending on the clinic. This results in very different practices in different clinics. In Westphalia-Lippe an average of 48 children (*N* = 1008/standard deviation = 90.8/range 1 to 1547) were examined per screener in 2016 [9].

### 3.5. Quality Requirements

The Children’s Directive [1] lists the following quality criteria for hospitals:

- At least 95% of children born in a hospital should undergo hearing screening (“coverage”).
- At least 95% of children who failed the initial screen should receive an AABR rescreening at the same hospital before being discharged.
- The proportion of children with findings requiring follow-up after being discharged from hospital (the “referral rate”) should not exceed 4%.

Data from the hearing screening project in Westphalia-Lippe [9], which has been active since 2007, shows that these criteria are very demanding. A coverage rate of greater than 95% was only achieved from 2010 onwards (2016, 5.67%). The referral rate has been stable for years, with an average of approximately 4% (2016, 3.8%).

### 3.6. Screening Devices

All devices allowing ATEOAE and/or AABR measurements are suitable as screening devices. The most common and suitable devices used in Germany are listed in Table 1. Since there is no central hearing screening center in Germany, various data transmission paths have been established by different hearing screening centres, some of which are directly supported by screening devices. One special feature of individual devices is bidirectional data transfer, which enables technical support of the devices by hearing screening centres and the extension of the NHS screening process using telemedical technology.

### 3.7. Service Funding for Inpatient Births

Newborns have a legal right to newborn hearing screening paid for by the health insurance company. This is included in the remuneration paid to the care provider for an inpatient birth according to the German reimbursement system for hospitals (German Diagnosis Related Groups). Calculated total costs (personnel, consumables, maintenance) amount to approximately 13–17 euros per examination [10]. This amount per measurement is a value comparable to international cost analyses [11,12,13], taking into account the total cost over several measurements of two- or three-stage screening.

### 3.8. Responsibility and Funding for Screen Tests outside the Hospital

If hearing screening is not carried out at the birth centre, it can be carried out as part of the child’s standard medical check-ups as defined in the Children’s Directive [1], by the child’s paediatrician or by specialists for speech, voice and childhood hearing disorders (phoniatricians and paediatric audiologists) or ear, nose and throat doctors specially trained in paediatric audiology.

The Institute of the Appraisal Committee [14], the body which oversees the remuneration system for contractual medical care in Germany, defines the calculable remuneration to the care provider for medical counselling and performing hearing screening in newborns in paragraphs 1704 to 1706 of the German Medical Fee Index (Gebührenordnung für Ärzte).

## 4. Follow-Up/Diagnostics

Minimum requirements for medical follow-up centres have been defined by the Ear, Nose and Throat (ENT) Association and the Association of Phoniatricians and Paediatric Audiologists (phoniatrics is the medical specialty for communication disorders in which the clinical role is similar to that of speech pathologists and speech and language therapists in other countries) and their respective scientific societies [15]. These requirements specify two types of follow-up sites (Level 1 and Level 1 + 2), which differ in their suitability and diagnostic possibilities.

Level 1 centres comprise established specialists from the above-mentioned specialties or corresponding clinics with adequate facilities, who are able to carry out the following examinations:

- ear microscopy/otoscopy
- tympanometry 226 Hz & 1000 Hz
- TEOAE
- diagnostic measurement of auditory brainstem responses (ABR) if necessary

Level 1 + 2 centres are characterised by their specialist pediatric audiological profile and a higher audiological qualification. These are established specialists from the above-mentioned specialties or corresponding clinics with adequate facilities, who can carry out the following examinations:

- ear microscopy/otoscopy
- tympanometry 226 Hz and 1000 Hz
- TEOAE, ideally with additional frequency specific measurement of distortion product otoacoustic emissions (DPOAE)
- frequency-specific ABR with hearing threshold estimation possible for at least 2 frequencies, ideally with click stimuli for latency assessment
- behavioural observation audiometry for ages 0−6 months as a check on the plausibility of objective audiometry results

In addition, where hearing impairment is diagnosed, level 1 + 2 centres are able to carry out or initiate and monitor the following early intervention steps:

- initiation and monitoring of hearing aid use
- counselling parents/carers
- carrying out or initiating an investigation into the etiology/cause of the hearing impairment
- initiation of early intervention support programmes
- further interdisciplinary diagnostics, if required

### Funding of the Follow-Up

As a rule, these diagnostic services are funded by health insurance companies.

## 5. Tracking

Where test results require follow-up, it is expected that the child and their parents/carers will attend a specialist clinic for medical follow-up as soon as possible, as described in Section 4. This is successful in most cases, but many children with findings that require monitoring do not attend follow-up at all (lost to follow-up) or attend very late [16]. The most challenging task facing NHS and hearing screening centers (HSCs) is not the earlier initiation of treatment of those who attend follow-up, but reducing the proportion of patients lost to follow-up.

**Figure 1:**
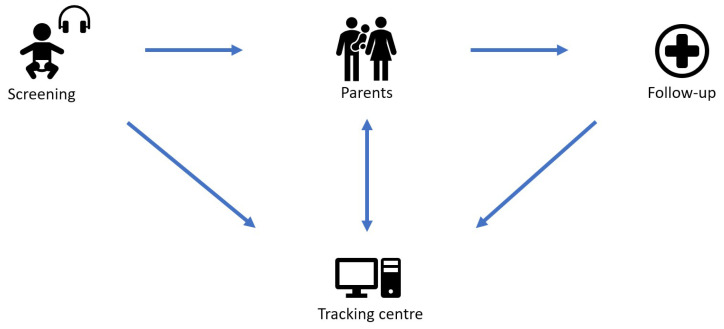
Information exchange in the NHS.

For this purpose, HSCs (or “tracking centres”) coordinate the exchange of information between maternity facilities, parents/carers and follow-up specialists, as shown schematically in Figure 1.

In order to ensure that the information from these sources (maternity clinics, follow-up centres and parents/carers) is correctly merged, many HSPs work with “screening IDs”, a unique number that is assigned to the child by the maternity ward.

### 5.1. Association of German Hearing Screening Centers (VDHZ e.V.)

**Figure 2:**
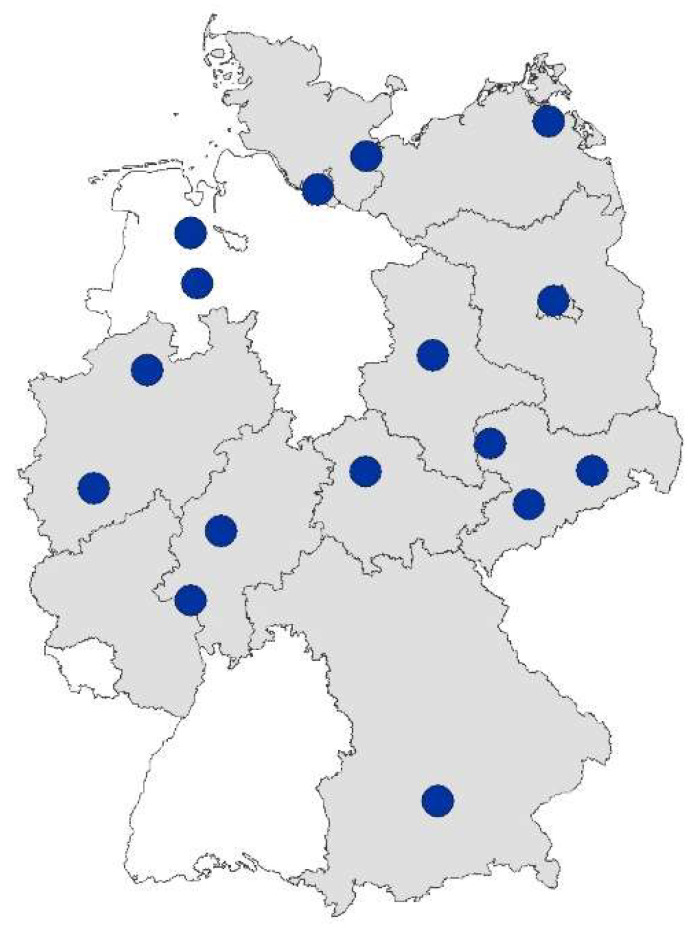
Hearing screening centers in Germany.

There are currently 16 hearing screening centers (HSCs) across 13 states in Germany (see overview map in Figure 2). They have joined forces to form the Association of German Hearing Screening Centers (VDHZ e.V.) and maintain a website with information on the NHS programme in Germany (www.vdhz.org).

The aim of the association is to promote newborn hearing screening through:

- Informing the public about the need for NHS, the methods used, possible results and treatment.
- Initiation and promotion of a nationwide network of hearing screening centres.
- Promotion of counselling, training and information for all persons involved in the NHS.
- Initiation and promotion of research projects and evaluation in the field of newborn hearing screening.

The creation of uniform definitions for comparative evaluations [17], support for the evaluation of the NHS on behalf of the Gemeinsamer Bundesausschuss (G-BA, the German Joint Federal Committee, which is the highest decision-making body of the joint self-government of physicians, hospitals and health insurance funds in Germany) [18], the provision of online benchmarking for birth clinics [19] and the provision of an infrastructure for developers of tracking software for tracking children across project boundaries [20] are concrete activities performed in cooperation with the association.

### 5.2. Information Exchange

In smaller, locally based projects, data exchange between the participants is possible with paper-based solutions or by telephone or e-mail. However, as the size of the project increases, these communication methods quickly reach their economic and organizational limits. Automatic, often modem- or IP-based solutions are therefore used by most HSCs today for the transfer of data between the maternity clinic and the HSC (screening results) and the follow-up clinics and the HSC (findings/diagnoses). HSCs are often quite complex and large telematic projects which require considerable additional work by staff members to check screening data, obtain addresses via inquiries at birth centres or registration offices, and sending reminder letters to parents/carers (more than 8000 letters per year in the Westfalen-Lippe HSC).

### 5.3. Structure, Costs and Financing of Hearing Screening Centers

In contrast to screening itself and specialist medical diagnostics, the Children’s Guideline makes no concrete statements about the structure and financing of the hearing screening centres. As a result, the financing and organisational structure is very different at each one, ranging from direct or indirect state support, financing through service contracts for quality assurance with the participating clinics, to combinations of the use of university resources together with donations or grants.

These structural and financial differences have practical implications for the coverage, completeness of data and endpoint of tracking. Coverage depends on whether collaboration with HSCs is mandatory or voluntary for all hospitals, a factor which differs from state to state in Germany. The completeness of the NHS is influenced amongst other things, by the ability to access and compare data held by the HSCs against a birth register or a connection to other early examinations (e.g., metabolic screening). The endpoint of screening (i.e., the step in the screening, diagnosis and therapy process up to which point data is gathered) is in turn influenced by financial and legal framework conditions.

Cost analysis of the hearing screening center in Westphalia-Lippe and data from Hessen [10] show that tracking (i.e., the work of the HSC) costs 3−4 Euros per child.

## 6. Evaluation

The German NHS programme was evaluated in accordance with the Children’s Guideline on behalf of the G-BA for the cohorts of children born in 2011 and 2012 [3]. The final report of this evaluation credits the NHS as having been successfully implemented overall.

When considering process quality, the main objectives of the NHS were not achieved. The proportion of screened children (82.4% in 2011 and 79.5% in 2012) did not reach the target of 95%. The referral rates in both years (both 5.3%) were above the required 4%. If one looks at the quality of the results, the targeted maximum age of diagnosis (three months) was unfortunately not reached (medians of four and five months respectively). The calculated prevalence of permanent, connatal, bilateral hearing impairment (greater than 20 dB HL) was comparable to other countries, at 1.3 per 1000 live births [21,22,23]. In all parameters there were large differences between the different states and also between regions with and without hearing screening centers.

Criticism was directed to the low percentage of cases in which the second stage of screening was undertaken following referral for a second test on the first test before discharge from hospital in comparison with the requirements of the Children’s Guideline. This leads to a high rate of children with findings requiring follow-up (referral rate) and therefore to a greater burden on the follow-up examination centers. One outcome from this evaluation was that the duration before diagnosis and initiation of therapy was significantly reduced with the introduction of nationwide newborn hearing screening. The considerable discrepancies in screening between regions in terms of the process, adherence to legal requirements, documentation and the definition of the required data set suggests that there is scope for improvement of all aspects of the NHS in Germany.

## 7. Problems and Opportunities

In federally-organised countries, such as Germany, the interrelation and overlap of healthcare and data protection responsibilities between the federal government and the individual states, as well as between healthcare providers and funders, can lead to difficulties around the availability of the necessary clinical competencies required for the NHS and problems at the intersections of cooperation. Despite data protection law existing at the federal level, individual states have formed different interpretations of it. This has had concrete effects on the extent and duration of data storage permitted within NHS. Data merging has also been made difficult due to different states having differently defined tracking endpoints and the large number of software packages being used for tracking (three commercial products and a number of local solutions are in use across the 16 hearing screening centres). The different structures and financing models of the various hearing screening centers have led to each having slightly different responsibilities and objectives. However, this has led to creative solutions being found to problems. The Association of German Hearing Screening Centers is trying to unify such issues, as already described above. This could be achieved by building consensus on uniform definitions (e.g., the definition of the target condition for which NHS is undertaken) or by providing IT solutions to interface problems.

The following section outlines one specific example of a problem which does not occur in centrally-organized projects or countries: tracking across project/state boundaries.

### Tracking across Project/State Boundaries

**Figure 3:**
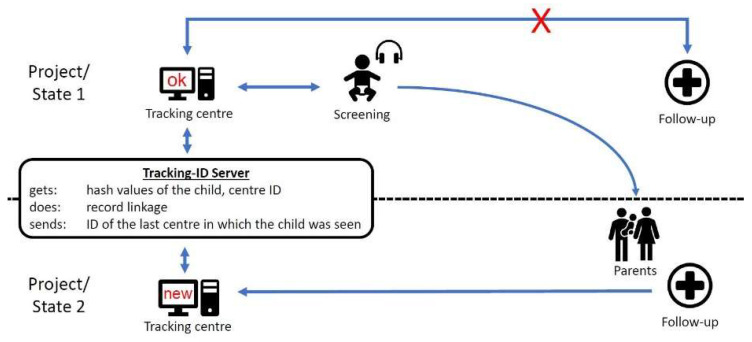
Tracking across project/state boundaries.

If a child has been screened and the result is “refer”, parents/carers are expected to contact a follow-up center. This is usually a facility near the maternity/birth clinic which cooperates with the screening center (Figure 3).

If the parents/carers attend a follow-up centre in another area of responsibility (because of parental choice or relocation), the initial tracking center will not receive any feedback from the follow-up centre and will therefore identify the child as “lost to follow-up”.

To solve this problem, a Tracking-ID server was developed [20] which allows all tracking centers to find the last centre that had contact with the family. It is important that this is done without the storage of any personal data of the child or the family, due to data protection regulations. The Tracking-ID-Server receives only hash values of some of the child’s demographics, the center ID and a timestamp. A record linkage using probabilistic methods is run and, in the case of a tracking centre searching for a lost child, the ID of the last centre in which the child was seen is sent (see Figure 3).

We hope that this solution will be integrated into the tracking software of the various hearing screening centers throughout the country.

## Figures and Tables

**Table 1:** Screening devices (selection).

Company/Holding	Natus	William Demand	Natus	Natus	Path Medical
**Division**	Otometrics	Maico			
**Unit**	Madson Accuscreen	MB11 BERAphone	echo-screen II	echo-screen III	Sentiero Advanced
**Device type**	Handheld	Handheld + Notebook	Handheld	Handheld	Handheld
**AABR**	Yes	Yes	Yes	Yes	Yes
**ATEOAE**	Yes	No	Yes	Yes	Yes
**Optional measurement methods**	DPOAE	Special ABR mode for follow-up	DPOAE	DPOAE	ABR, ASSR, DPOAE, FMDPOAE and others for follow-up
**Data transfer to the screening center**	Yes Directly out of the device with UMTS/LTE modem	Yes Email attachments with additional audio_cl software (Windows)	Yes Directly out of the device with UMTS/LTE modem or with audble lite software (Windows) and cloud services	Yes With audble lite software (Windows) and cloud services	Yes Directly out of the device with UMTS/LTE modem. For Follow-up: Attendance, Reassessment, Results, Diagnoses
**Data transfer to the device**	Yes User settings, Profiles, Updates, Reconfiguration after maintenance	No	No (only allowed within scientific projects)	No	Yes User settings, Profiles, Updates, Loan-device management for annual service and maintenance

Abbreviations: AABR = automated auditory brainstem response, ABR = auditory brainstem response, ASSR = auditory steady-state response, ATEOAE = automated transient-evoked otoacoustic emission, DPOAE = distortion product otoacoustic emission, FMDPOAE = frequency-modulated distortion product otoacoustic emission, LTE = long-term evolution, UMTS = universal mobile telecommunications system.
